# Drying Kinetics, Apparent Moisture Diffusivity, and Energy Performance of Black Soldier Fly Larvae in a Rotating-Tray Hot-Air Dryer

**DOI:** 10.3390/insects17100984

**Published:** 2026-09-23

**Authors:** Aphichon Mungchu, Sarawut Saenkham, Anuwat Saenpong, Chinnapat Turakarn, Sopa Cansee

**Affiliations:** 1 Department of Mechanical and Mechatronics Engineering, Faculty of Engineering and Industrial Technology, Kalasin University, Kalasin 46000, Thailand; aphichon.mu@ksu.ac.th (A.M.);; 2 Faculty of Engineering, Mahasarakham University, Kantharawichai 44150, Maha Sarakham, Thailand

**Keywords:** *Hermetia illucens*, insect biomass, convective drying, rotating tray, apparent moisture diffusivity, specific energy consumption

## Abstract

Fresh black soldier fly larvae (BSFL) have a high moisture content that complicates storage and requires energy-intensive postharvest drying. This study evaluated the effects of drying-air temperature and tray rotation speed on BSFL drying kinetics, energy performance, and moisture transport in a rotating-tray hot-air dryer. A 3 × 3 factorial experiment was conducted at 50, 60, and 70 °C and tray rotation speeds of 0, 5, and 10 rpm, with three independent replicates, while drying-air velocity was maintained at 3.0 m s^−1^. Drying time decreased from 31.0 h at 50 °C and 0 rpm to 6.5 h at 70 °C and 10 rpm. The latter condition achieved the highest drying efficiency (49.11%) and lowest specific energy consumption (4.60 MJ kg^−1^ water removed). Comparable values were obtained at 60 °C and 10 rpm (49.07% and 4.61 MJ kg^−1^ water removed, respectively). Among nine thin-layer models evaluated for the six rotating-tray treatments at 5 and 10 rpm, the Midilli model provided the best fit. Apparent effective moisture diffusivity ($D_{eff})$ increased from 0.2457 × 10^−9^ to 1.2946 × 10^−9^ m^2^ s^−1^, while apparent activation energy ranged from 67.39 to 68.46 kJ mol^−1^. Rotating-tray drying accelerated BSFL dehydration and improved engineering performance; however, microbial safety and product quality require separate evaluation before food or feed recommendations can be made.

## Simple Summary

Black soldier fly larvae contain high levels of moisture after harvesting and therefore require drying before further processing or extended storage. However, conventional hot-air drying can require long processing times and considerable electrical energy. This study evaluated a rotating-tray hot-air dryer operated at temperatures of 50, 60, and 70 °C and tray rotation speeds of 0, 5, and 10 revolutions per minute, while maintaining the drying-air velocity at 3.0 m s^−1^. Increasing the drying temperature substantially shortened the drying process, while tray rotation further reduced drying time and total energy consumption. The shortest drying time was 6.5 h at 70 °C and 10 revolutions per minute. This condition also produced the highest drying efficiency and lowest specific energy consumption. Nevertheless, drying at 60 °C and 10 revolutions per minute achieved nearly equivalent energy performance at a lower temperature. These findings demonstrate that controlled tray rotation can improve the drying and energy performance of hot-air drying systems for insect biomass. However, microbiological safety, nutritional retention, and physicochemical product quality were not evaluated; therefore, the investigated conditions should not be interpreted as an overall optimization for food or feed production.

## 1. Introduction

Growing demand for animal-derived protein has increased pressure on conventional feed-production systems and intensified competition for land, water, energy, and protein-rich feed ingredients. Consequently, insects have attracted interest as alternative feed resources because of their rapid growth, efficient biomass conversion, and ability to utilize low-value organic materials [1,2,3]. Among commercially reared insects, the black soldier fly, *Hermetia illucens* (L.) (Diptera: Stratiomyidae), is particularly promising because its larvae can convert diverse organic materials into biomass rich in protein and lipids [4,5,6,7]. This bioconversion capacity connects organic-waste management with animal-feed production and supports circular-bioeconomy strategies in which nutrients are recovered from residual materials and returned to productive food and feed systems [8,9,10,11]. However, the substrates permitted for rearing insects intended for food or feed depend on the applicable national or regional regulatory framework. In the European Union, for example, catering waste and manure are prohibited as substrates for farmed insects intended for feed production [12,13]. Therefore, the capacity of BSFL to utilize organic residues should be distinguished from the regulatory eligibility of the resulting biomass for commercial food or feed applications. Dried BSFL meal has long been investigated as a supplementary protein source in livestock diets, including swine production [14]. However, the sustainability of black soldier fly larvae (BSFL) production depends not only on rearing efficiency but also on the resource and energy requirements of postharvest processing.

Postharvest drying is an energy-intensive operation that can substantially affect the economic and environmental performance of biological-material processing [15]. Fresh BSFL generally contain more than 60% moisture on a wet basis, making them susceptible to rapid microbial and biochemical deterioration after harvesting [16,17]. Their high moisture content complicates storage, increases transportation costs, and complicates subsequent operations such as grinding, oil extraction, and feed formulation. Drying is therefore commonly applied to reduce moisture content and potentially limit deterioration during subsequent handling and storage, while facilitating transportation and conversion into insect meal or other processed materials. Inefficient drying may require prolonged operation and excessive energy input, thereby diminishing some of the resource-efficiency benefits achieved during larval rearing. Excessive thermal exposure may also alter heat-sensitive constituents, including proteins and lipids, although the magnitude of these changes depends on drying temperature, exposure time, and drying method [16,17]. Experimental modeling and optimization approaches have been applied to biological materials to relate operating conditions to processing responses and support operating-condition selection [18]. Developing a rapid and energy-efficient drying process is therefore important for the effective processing of BSFL biomass; however, drying and energy indicators alone cannot establish microbial safety or nutritional quality.

Several technologies have been investigated for drying insect biomass. Freeze drying can produce a porous and stable dried material, but its long processing time, high capital cost, and substantial energy demand generally limit its application at large production scales. Microwave and microwave-convective drying can accelerate moisture removal and reduce processing time; however, heating uniformity, process control, energy use, and product-quality retention remain important considerations [19]. Dielectric drying technologies, including microwave and radio-frequency drying, have also been evaluated for their effects on the microbiological quality, safety, and stability of BSFL [20]. Infrared drying has been applied to BSFL to accelerate moisture removal and evaluate the associated energy-performance responses [16]. In contrast, convective hot-air drying is widely used because of its operational simplicity, controllability, and suitability for batch or continuous processing [15,20]. Nevertheless, conventional static-tray drying may provide uneven exposure of densely distributed larvae to the drying air, resulting in localized moisture accumulation, prolonged drying, and elevated specific energy consumption. Increasing the drying-air temperature can accelerate water removal by increasing the thermal driving force, but higher temperatures may also increase thermal exposure and potentially affect heat-sensitive constituents [21]. Mechanical movement of the product, including tray rotation or agitation, may repeatedly change the material’s position relative to the airflow and thereby influence external heat and mass transfer.

Despite these potential advantages, limited quantitative information is available on the combined effects of drying-air temperature and tray rotation speed on the drying kinetics and energy performance of BSFL. Previous research has demonstrated the value of integrating drying-kinetics analysis, mathematical modeling, and energy-performance assessment for biological materials [22], while BSFL-specific studies have characterized convective drying kinetics and moisture-transport behavior [23]. Other studies have focused on individual drying variables, alternative heating methods, or final product characteristics [15,19,24], with less attention given to factorial interactions between drying-air temperature and mechanical tray rotation.

Furthermore, few studies have simultaneously separated the electrical energy consumed by the heater, blower, and tray-rotation system; determined drying efficiency (*η*) and specific energy consumption (*SEC*); evaluated thin-layer drying models; and estimated apparent effective moisture diffusivity ($D_{eff})$ and apparent activation energy. Such integrated information is needed to distinguish the contributions of thermal input and mechanical movement and to support process prediction, dryer design, and engineering-based operating-condition selection. The novelty of the present study lies in its integrated assessment of drying kinetics, component-level energy consumption, factorial effects, thin-layer model performance, and apparent moisture-transport behavior using a rotating-tray hot-air dryer with a practical batch capacity of 12 kg.

Accordingly, this study investigated the effects of drying-air temperature and tray rotation speed on the engineering performance of a rotating-tray hot-air drying system for BSFL. Drying-air velocity was maintained at 3.0 m s^−1^ throughout the experiments. Accordingly, this study investigated the effects of drying-air temperature and tray rotation speed on the engineering performance of a rotating-tray hot-air drying system for BSFL. Drying-air velocity was maintained at 3.0 m s^−1^ throughout the experiments. The specific objectives were to (i) evaluate moisture-ratio profiles, drying-rate behavior, and drying time under combined drying-air temperature and tray-rotation conditions; (ii) quantify the electrical energy consumed by the heater, blower, and tray-rotation motor and determine total energy consumption, *η*, and *SEC*; (iii) compare nine thin-layer drying models for the six rotating-tray treatments and identify the model providing the best fit to the experimental moisture-ratio data; and (iv) estimate apparent $D_{eff}$ and apparent activation energy as indicators of moisture-transport behavior and temperature sensitivity. The findings provide an engineering basis for comparing drying time and energy utilization under the investigated operating conditions. Microbiological lethality, water activity, nutrient retention, protein digestibility, lipid oxidation, and commercial food/feed suitability were not evaluated; accordingly, the conclusions are restricted to drying process and energy performance.

## 2. Materials and Methods

### 2.1. Black Soldier Fly Larvae and Sample Preparation

Fresh BSFL, *Hermetia illucens* (L.) (Diptera: Stratiomyidae), were obtained from a local producer in Kantharawichai District, Maha Sarakham Province, Thailand. The larvae were reared on mixed food waste collected from a local canteen and harvested at the late larval stage. The producer supplied and managed the substrate; ingredient proportions and proximate composition were not standardized or analytically determined. The exact chronological age of the larvae was not available; therefore, the late larval stage was identified from their external morphological characteristics. Upon receipt, larval viability was assessed manually by observing spontaneous movement and movement in response to gentle mechanical stimulation. Only live, active, and apparently healthy individuals of approximately uniform size were selected. Residual rearing substrate, frass, and other impurities were removed by sieving, after which the larvae were thoroughly rinsed with clean water.

Before drying, the larvae were blanched in boiling water at approximately 100 °C for 10–15 s to inactivate them and provide a standardized pretreatment before drying. Blanching has previously been reported to reduce the microbial load of insect biomass [25]; however, microbial counts were not determined in the present study. Following blanching, the samples were drained at room temperature until no visible surface water remained and subsequently stored at 4 °C until the drying experiments. The initial moisture content was determined in triplicate using the oven-drying method described in ISO 1442:1973 [25]. Approximately 5 g of larvae was weighed and dried in a laboratory oven at 105 °C for 72 h or until a constant mass was attained. The initial moisture content was 64.83 ± 2.00% on a wet basis (w.b.).

### 2.2. Rotating-Tray Hot-Air Drying System

A rotating-tray hot-air dryer was designed and constructed to improve BSFL exposure to the drying air. The system comprised a thermally insulated drying chamber, an electric heating unit, a forced-air circulation system, six circular trays arranged horizontally at vertically separated levels, and an electrical control and measurement unit. The horizontal-tray configuration was selected to maintain a defined sample loading on each tray while periodically changing the samples’ position relative to the air-distribution slots. A rotating-drum configuration, in which the samples would undergo continuous tumbling, was not evaluated because it represents a different contact and mixing mechanism that would require a separate experimental design. Figure 1 schematically illustrates the structural configuration, airflow path, tray-drive assembly, and electrical control system. Supplementary Table S1 summarizes the instruments used to monitor mass, air velocity, temperature, and electrical energy.

#### 2.2.1. Drying Chamber and Heating Unit

The drying chamber was constructed from SUS 304 stainless steel and thermally insulated to reduce heat loss to the surrounding environment. The chamber measured 99 cm wide, 70 cm deep, and 122 cm high, with an insulated wall thickness of 5 cm. The front access door measured 76 cm × 128 cm and was also insulated with a thickness of 5 cm to limit heat loss during operation.

An electric finned-tube heater supplied thermal energy with a nominal power rating of 3.0 kW. The heater operated using a 240 V, 50 Hz electrical supply and was installed upstream of the drying chamber. The finned-tube configuration provided an extended heat-transfer surface for heating the circulating air before it entered the chamber. The temperature-control system regulated the drying-air temperature to the selected set point of 50, 60, or 70 °C, as shown in Figure 1b.

#### 2.2.2. Hot-Air Circulation and Temperature Control

Hot air was circulated through two 75 mm diameter ducts constructed from SUS 304 stainless steel. A centrifugal blower with a 20 cm impeller generated air movement, driven by a 250 W electric motor operating at 220 V and 50 Hz. The blower operated at 2800 rpm and provided a nominal volumetric airflow rate of 504 m^3^ h^−1^.

Within the drying chamber, 12 rectangular air-distribution slots, each measuring 4 cm × 56 cm, were arranged symmetrically along both side walls. This arrangement was designed to distribute the heated air across the tray surfaces.

The drying-air velocity could be adjusted within the range of 3–6 m s^−1^; however, it was maintained at 3.0 m s^−1^ across all experimental treatments and was independent of the tray rotation speeds of 0, 5, and 10 rpm. Drying-air velocity was measured using a hot-wire anemometer.

Drying-air temperature was monitored using K-type thermocouples connected to a multichannel data logger. The thermocouples were positioned at the chamber center. The heating unit was controlled to maintain the drying chamber at the designated experimental temperature.

#### 2.2.3. Rotating-Tray and Drive System

Six circular drying trays, each 60 cm in diameter, were arranged horizontally at vertically separated levels within the drying chamber. The trays were connected to a common vertical central shaft and rotated in the horizontal plane.

The trays were fabricated from stainless steel, with a mesh size of 6 mm, and connected to a central drive assembly. A geared drive mechanism mounted at the top of the chamber transmitted rotational motion through a vertical central shaft, enabling the horizontally arranged trays to rotate at 0, 5, or 10 rpm. Based on the tray diameter of 0.60 m, these rotation rates corresponded to peripheral speeds of 0, 0.157, and 0.314 m s^−1^ at the tray edge, respectively. During each experimental run, 2 kg of BSFL was uniformly distributed on each tray, giving a total dryer loading of 12 kg. Tray rotation was designed to vary the position of the samples relative to the air-distribution slots, thereby promoting repeated exposure to the circulating hot air. However, because spatial moisture uniformity was not directly measured, the system is described as being designed to improve air exposure rather than being confirmed to produce uniform drying.

#### 2.2.4. Electrical Energy Measurement

The electrical energy consumed by the heater, blower, and tray-drive motor was determined separately during each drying experiment. Electrical power was monitored using an AC power meter, while the operating duration of each component was recorded throughout the drying process. The energy consumption of each component was calculated from its measured electrical power and operating time. Total electrical energy consumption was determined as the sum of the energy consumed by the heater, blower, and tray-drive motor. The corresponding equations for total energy consumption, *η*, and *SEC* are presented in Section 2.8.

### 2.3. Experimental Design and Drying Procedure

A 3 × 3 full factorial design was employed to evaluate the individual and interactive effects of drying-air temperature and tray rotation speed on the drying kinetics and energy performance of BSFL. The experimental factors comprised three drying-air temperatures (50, 60, and 70 °C) and three tray rotation speeds (0, 5, and 10 rpm), resulting in nine treatment combinations. A rotation speed of 0 rpm represented the stationary-tray control. The drying-air velocity was maintained at 3.0 m s^−1^ throughout all experiments and was independent of the applied tray rotation speed. Each treatment combination was conducted in three independent experimental runs, and the run order was randomized. The selected temperature range was consistent with that commonly investigated for the convective drying of insects and other heat-sensitive biological materials [17,23]. The temperatures of 50, 60, and 70 °C represented three systematically spaced levels within a practical convective-drying range and permitted evaluation of temperature-dependent changes in drying kinetics, apparent moisture diffusivity, processing time, and energy performance. These temperatures were not selected to establish microbial lethality, nutrient preservation, or an overall food/feed-quality optimum because these responses were outside the experimental design.

For each experimental run, BSFL with an initial moisture content of approximately 64.8% w.b. were evenly distributed over the surfaces of six drying trays. Each tray was loaded with 2 kg of BSFL, resulting in a total batch mass of 12 kg per run. The dryer was operated at the designated combination of drying-air temperature and tray rotation speed. Sample mass was recorded at 30 min intervals, and drying was continued until the calculated moisture content reached approximately 10% w.b., which was used as a consistent engineering endpoint for comparing drying time and energy performance among treatments [24]. Because water activity and microbiological quality were not measured, reaching this moisture content was not interpreted as confirmation of storage stability or microbial safety. Electrical energy use was monitored throughout each experimental run. The energy consumed by the heater, blower, and tray-drive motor was determined separately from the measured electrical power and corresponding operating time. Total electrical energy consumption was calculated by summing the measured energy consumption of these components. This energy-accounting approach and the resulting measurements were used to calculate *η* and *SEC*, consistent with established drying-energy analyses [26], as detailed in Section 2.8.

### 2.4. Drying Characteristics

#### 2.4.1. Moisture Content

The drying characteristics of BSFL were evaluated by recording changes in sample mass at 30 min intervals during rotating-tray hot-air drying. Moisture content was expressed on both wet and dry bases. Wet-basis moisture content was used to determine whether the samples reached the predefined engineering endpoint, whereas dry-basis moisture content was used to calculate the moisture ratio and drying rate. Moisture contents on wet and dry bases were calculated using Equations (1) and (2), respectively, following the gravimetric principles described in ISO 1442:1973 [25] and standard drying-analysis procedures [27,28]:

(1) $$X_{t,wb}=\frac{w_{t}-w_{d}}{w_{t}}\;\times 100$$

where $X_{t,wb}$ is the moisture content at drying time t (% w.b.), $w_{t}$ is the sample mass at time t (kg), and $w_{d}$ is the oven-dried solid mass (kg).

For drying-kinetics analysis, moisture content on a dry basis was calculated using Equation (2):

(2) $$X_{t,db}=\frac{w_{t}-w_{d}}{w_{d}}$$

where $X_{t,db}$ is the moisture content at drying time t, expressed as kg water kg^−1^ dry matter. Wet-basis moisture content was used to determine when the BSFL reached the predefined endpoint of approximately 10% w.b. This endpoint was used for consistent comparison among treatments and was not interpreted as confirmation of storage stability or microbiological safety.

#### 2.4.2. Moisture Ratio

To normalize the drying curves across the experimental treatments, the moisture ratio was calculated using Equation (3), following Akpinar [27] and Onwude et al. [28]:

(3) $$MR=\frac{X_{t,db}-X_{e,db}}{X_{o,db}-X_{e,db}}$$

where MR is the dimensionless moisture ratio, $X_{t,db}$ is the dry-basis moisture content at drying time t, $X_{o,db}$ is the initial dry-basis moisture content, and $X_{e,db}$ is the equilibrium moisture content, all expressed as kg water kg^−1^ dry matter. When equilibrium moisture content is small relative to the initial moisture content, the general moisture-ratio expression can be simplified without substantially affecting thin-layer model fitting, as shown in Equation (4) [27,28]:

(4) $$MR=\frac{X_{t,db}}{X_{o,db}}$$

Equation (4) was used for thin-layer model fitting because equilibrium moisture content was not independently determined. Accordingly, the estimated model coefficients were interpreted as apparent empirical parameters under the investigated drying conditions.

#### 2.4.3. Drying Rate and Drying Time

The drying rate was calculated from the change in dry-basis moisture content between two consecutive measurements using Equation (5) [29]:

(5) $$DR\;=\frac{X_{t,db}-X_{t+\Delta t,db}}{\Delta t}$$

where DR is the drying rate (kg water kg^−1^ dry matter h^−1^), $X_{t,db}$ is the moisture content at time t, $X_{t+\Delta t,db}$ is the moisture content at the subsequent measurement time, and Δt is the measurement interval (h). In this study, Δt=0.5 h because sample mass was recorded every 30 min. Drying time was operationally defined using Equation (6) as the elapsed time from the beginning of drying until the sample first reached the predefined engineering endpoint of approximately 10% w.b.:

(6) $$t_{d}\;=t_{f}-t_{0}$$

where $t_{d}$ is the drying time (h), $t_{0}$ is the starting time (h), and $t_{f}$ is the time at which the BSFL first reached approximately 10% w.b. Because $t_{0}=0$, $t_{d}=t_{f}$. The reported drying-time resolution was 0.5 h. The endpoint was used to compare drying-process performance and did not verify water activity, microbial stability, or commercial storage suitability.

### 2.5. Thin-Layer Drying Modeling

#### 2.5.1. Thin-Layer Models

The experimental moisture-ratio data were fitted to nine thin-layer drying models: Midilli [30], Wang and Singh [31], approximation of diffusion [32], Page [33], logarithmic [34], two-term [35], modified Henderson and Pabis [36], Henderson and Pabis [37], and Newton [38]. Supplementary Table S2 summarizes the mathematical expressions and original references for these models. Model parameters were estimated separately for the six rotating-tray treatments comprising three drying-air temperatures (50, 60, and 70 °C) and two tray rotation speeds (5 and 10 rpm). The three stationary-tray treatments at 0 rpm were retained as control conditions for evaluating the effects of tray rotation on drying time, drying-rate behavior, energy performance, and apparent effective moisture diffusivity but were not included in the detailed thin-layer model comparison. Parameter estimation was performed by minimizing the sum of squared differences between the experimental and model-predicted moisture-ratio values.

#### 2.5.2. Model-Selection Criteria

The best-fitting model was evaluated using the coefficient of determination ($R^{2}$), reduced chi-square (χ2), and root mean square error (*RMSE*). These statistical indicators are widely used to assess agreement between the experimental and predicted moisture-ratio data [17,27,39] and were calculated using Equations (7)–(9):

(7) $$R^{2}\;=\;1\;-\;\left(\frac{\sum_{i=1}^{N}{\left(MR_{exp,i}-MR_{pred,i}\right)}^{2}}{\sum_{i=1}^{N}{\left(MR_{exp,i}-{\bar{MR}}_{exp}\right)}^{2}}\right)$$

(8) $$\chi^{2}=\frac{\sum_{i=1}^{N}{\left(MR_{exp,i}-MR_{pred,i}\right)}^{2}}{N-z}$$

(9) $$\mathrm{RMSE}={\left(\frac{1}{N}\sum_{i=1}^{N}{\left(MR_{exp,i}-MR_{pred,i}\right)}^{2}\right)}^{1/2}$$

where *MRexp,i* and *MRpred,i* are the experimental and model-predicted moisture ratios of the *ith* observation, respectively; ${\bar{MR}}_{exp}$ is the mean experimental moisture ratio; *N* is the total number of observations; and *z* is the number of independently estimated model parameters. The most appropriate model was selected as that providing the highest $R^{2}$ and the lowest *RMSE* and *χ*^2^ values. Model selection was conducted separately for the six rotating-tray treatments to determine whether the best-performing model remained consistent across drying-air temperatures and tray rotation speeds. Accordingly, model selection and validation were restricted to the six treatments at 5 and 10 rpm and did not include the stationary 0 rpm controls.

### 2.6. Apparent Effective Moisture Diffusivity Estimation of Apparent Effective Moisture Diffusivity

Apparent effective moisture diffusivity ($D_{eff}$) was estimated using the analytical solution of Fick’s second law of diffusion. Individual BSFL were approximated as infinite slabs with uniform initial moisture distribution. The characteristic half-thickness, L, was calculated as half of the mean larval thickness $\left(\delta \right)$ using Equation (10):

(10) $$L=\;\frac{\delta }{2}$$

The mean larval thickness was 10 ± 0.15 mm, determined from 50 randomly selected larvae using a Vernier caliper. Measurements were performed immediately before blanching, when the samples were at the late larval stage. The producer did not provide the larvae’s exact chronological age. The diffusion analysis assumed one-dimensional moisture transport, constant effective diffusivity during each drying treatment, negligible external mass-transfer resistance, and negligible changes in larval geometry and shrinkage during drying. Accordingly, the calculated $D_{eff}$ values represent apparent effective diffusivities under the investigated drying conditions.

For an infinite slab, the dimensionless moisture ratio is expressed using Equation (11) [24,32]:

(11) $$MR=\frac{8}{\pi^{2}}\sum_{n=0}^{\infty }\frac{1}{{\left(2n+1\right)}^{2}}exp\left[-\frac{{\left(2n+1\right)}^{2}\pi^{2}D_{eff}t}{4L^{2}}\right]$$

where *MR* is the dimensionless moisture ratio, $D_{eff}$ is the apparent effective moisture diffusivity (m^2^ s^−1^), t is drying time (s), L is the characteristic half-thickness of the larvae (m), and n is a non-negative integer. For sufficiently long drying periods, the higher-order terms become negligible; therefore, only the first term (n=0) was retained, yielding Equation (12) [24,32]:

(12) $$MR=\frac{8}{\pi^{2}}exp\left(-\frac{\pi^{2}D_{eff}t}{4L^{2}}\right)$$

Taking the natural logarithm of Equation (12) yields Equation (13):

(13) $$ln(MR)=\;ln(\frac{8}{\pi^{2}})-\frac{\pi^{2}D_{eff}}{4L^{2}}t$$

The slope, $K_{d}$, of the linear relationship between ln(MR) and drying time was therefore calculated using Equation (14):

(14) $$K_{d}=-\frac{\pi^{2}D_{eff}}{{4L}^{2}}$$

Consequently, $D_{eff}$ was calculated using Equation (15):

(15) $$D_{eff}=-\frac{4L^{2}K_{d}}{\pi^{2}}$$

Linear regression of ln(*MR*) against drying time was performed separately for each combination of drying-air temperature and tray rotation speed. Drying time was converted from hours to seconds before calculating apparent $D_{eff}$. The detailed apparent $D_{eff}$ calculations and Arrhenius regression parameters are provided in Supplementary Table S3. Because $D_{eff}$ was derived from overall drying curves using simplified geometric and transport assumptions, it should not be interpreted as an intrinsic molecular-diffusion coefficient of BSFL tissue.

### 2.7. Activation Energy

The temperature dependence of $D_{eff}$ was described using an Arrhenius-type relationship presented in Equation (16) [24,32]:

(16) $$D_{eff}=D_{0}e^{\left(-\frac{E_{a}}{R\;T}\right)}$$

where $D_{0}$ is the pre-exponential factor (m^2^ s^−1^), $E_{a}$ is the activation energy (kJ mol^−1^), *R* is the universal gas constant (0.008314 kJ mol^−1^ K^−1^), and *T* is the absolute drying-air temperature (K).

Equation (16) was linearized to obtain Equation (17):

(17) $$ln(D_{eff})=\;ln(D_{0})-\frac{E_{a}}{R}(\frac{1}{T})$$

The activation energy was obtained from the slope, $K_{a}$, of the linear regression of $ln\left(D_{eff}\right)$ against 1/T, as expressed in Equation (18):

(18) $$K_{a}=\;-\frac{E_{a}}{R}$$

Therefore, the activation energy was calculated using Equation (19)

(19) $$E_{a}=-K_{a}R$$

The Arrhenius analysis was performed separately at 0, 5, and 10 rpm to determine whether tray rotation affected the apparent energy barrier for moisture transport under the investigated conditions. Because each Arrhenius regression was based on three drying-air temperatures, the resulting activation energies were interpreted as apparent estimates within the evaluated temperature range.

### 2.8. Energy Performance

#### 2.8.1. Component and Total Electrical Energy Consumption

Electrical energy consumption was determined separately for the heater, blower, and tray-rotation motor. Electrical power was monitored throughout each drying run, and the energy consumed by each component was calculated from its power demand and operating duration. When power varied during operation, particularly because of heater cycling, component energy consumption was calculated by summing the electrical energy used over successive measurement intervals, as expressed in Equations (20)–(22) [26].

(20) $$E_{h}=\sum_{j=1}^{N}P_{h,j}{∆t}_{j}$$

(21) $$E_{b}=\sum_{j=1}^{N}P_{b,j}{∆t}_{j}$$

(22) $$E_{r}=\sum_{j=1}^{N}P_{r,j}{∆t}_{j}$$

where $E_{h}$, $E_{b}$, and $E_{r}$ are the electrical energies consumed by the heater, blower, and tray-rotation motor, respectively (kWh); $P_{h,j}$, $P_{b,j}$, and $P_{r,j}$ are the corresponding electrical power demands during the *jth* measurement interval (kW); ${∆t}_{j}$ is the duration of the measurement interval (h); and N is the total number of measurement intervals. For the stationary-tray treatment at 0 rpm, $E_{r}=0$.

Total electrical energy consumption was calculated as the sum of the energy consumed by each component, as expressed in Equation (23):

(23) $$E_{total}=E_{h}+E_{b}+E_{r}$$

where $E_{total}$ is the total measured electrical energy consumption (kWh). For the calculation of *η* and *SEC*, total electrical energy was converted from kWh to MJ using Equation (24):

(24) $$Q_{total}=3.6E_{total}$$

where $Q_{total}$ is the total electrical energy input (MJ), based on the conversion 1 kWh =3.6 MJ.

The mass of water removed during drying was calculated using Equation (25):

(25) $$W_{d}=W_{i}-W_{f}$$

where $W_{d}$ is the mass of water removed (kg), $W_{i}$ is the initial sample mass (kg), and $W_{f}$ is the final sample mass (kg). Because the dry-solid mass remained unchanged during drying, the reduction in sample mass was attributed to moisture removal.

#### 2.8.2. Drying Efficiency

Drying efficiency (*η*) was defined as the ratio of the theoretical energy required to evaporate the removed water to the total electrical energy supplied to the drying system and was calculated using Equation (26) [26,40]:

(26) $$\eta =\frac{W_{d}{\times \;h}_{fg}}{Q_{total}}\times 100$$

where *η* is the drying efficiency (%), $W_{d}$ is the mass of water removed (kg), ${\;h}_{fg}$ is the latent heat of vaporization of water (2.257 MJ kg^−1^), and $Q_{total}$ is the total electrical energy input (MJ).

#### 2.8.3. Specific Energy Consumption

Specific energy consumption (*SEC*) was calculated as the total electrical energy input required to remove a unit mass of water and was calculated using Equation (27) [41]:

(27) $$SEC=\frac{Q_{total}}{W_{d}}$$

where SEC is the specific energy consumption (MJ kg^−1^ water removed), $Q_{total}$ is the total electrical energy input (MJ), and $W_{d}$ is the mass of water removed (kg).

### 2.9. Statistical Analysis

The experimental unit was one complete drying run containing 12 kg of BSFL distributed across six trays. Each of the nine treatment combinations was independently replicated three times, resulting in 27 experimental runs. The results are presented as mean ± standard deviation. The effects of drying-air temperature, tray rotation speed, and their interaction were evaluated using two-way analysis of variance (ANOVA). When significant effects were detected, treatment means were compared using Tukey’s honestly significant difference (HSD) test at *p* < 0.05. All analyses were performed using IBM SPSS Statistics, version 29.0 (IBM Corp., Armonk, NY, USA).

## 3. Results

### 3.1. Moisture-Ratio Profiles and Drying Time

The initial moisture content of the fresh BSFL was 64.83 ± 2.00% w.b., corresponding to an initial *MR* of 1.00. Moisture ratio was calculated using Equation (4). Drying was continued to the predefined engineering endpoint of approximately 10% w.b.

Figure 2 presents the resulting *MR* profiles for the nine combinations of drying-air temperature and tray rotation speed. Each plotted value represents the mean of three independent experimental runs, and the error bars indicate ± SD. Moisture ratio decreased continuously with drying time under all experimental conditions. At 50 °C, the mean drying times were 31.0, 27.5, and 26.5 h at 0, 5, and 10 rpm, respectively (Figure 2a). Increasing the temperature to 60 °C reduced the corresponding drying times to 16.0, 11.0, and 10.0 h (Figure 2b). The shortest drying times were observed at 70 °C, with values of 8.0, 7.0, and 6.5 h at 0, 5, and 10 rpm, respectively (Figure 2c).

At each rotation speed, increasing the drying-air temperature substantially shortened the drying time. Tray rotation also reduced drying time at each temperature, although the magnitude of this reduction varied with drying-air temperature. At 50 °C, increasing the rotation speed from 0 to 10 rpm shortened drying time by 4.5 h. The corresponding reductions were 6.0 h at 60 °C and 1.5 h at 70 °C. Differences between 5 and 10 rpm were comparatively small, with drying-time reductions of 1.0, 1.0, and 0.5 h at 50, 60, and 70 °C, respectively.

### 3.2. Drying-Rate Characteristics

The drying rate (*DR*), calculated using Equation (5), was plotted against *MR* for the different drying-air temperatures and tray rotation speeds, as presented in Figure 3. Each plotted value represents the mean of three independent experimental runs, and the error bars indicate ±SD. The drying rate was highest during the initial stage, when *MR* was high, and progressively decreased as *MR* declined. No distinct constant-rate period was observed, indicating that BSFL drying occurred predominantly in the falling-rate period.

At 50 °C, the drying-rate profiles differed only slightly among the three rotation speeds (Figure 3a). Rotation produced more pronounced differences in drying rate at 60 and 70 °C, particularly during the early drying stage at high *MR* values (Figure 3b,c). At these temperatures, the 10 rpm treatment generally exhibited the highest drying rate, followed by the 5 and 0 rpm treatments. However, the drying-rate profiles at 5 and 10 rpm became increasingly similar as *MR* decreased, indicating only a small additional increase in drying rate associated with rotation at 10 rpm compared with 5 rpm during the later drying stage. These drying-rate patterns describe the overall treatment responses; because tray-to-tray and within-tray moisture distributions were not measured, they should not be interpreted as evidence that rotation improved drying uniformity.

### 3.3. Electrical Energy Consumption

The electrical energy consumed by the heater, blower, and tray-rotation motor under the nine drying conditions is summarized in Table 1. The heater accounted for the largest share of total electrical energy consumption, ranging from 7.500 to 12.400 kWh. Blower energy consumption ranged from 0.975 to 4.650 kWh and decreased as drying time shortened. The tray-rotation motor consumed 0.140–0.550 kWh at 5 rpm and 0.228–0.928 kWh at 10 rpm, whereas no rotation energy was consumed under the stationary-tray condition. At 50 °C, total electrical energy consumption decreased from 17.050 kWh at 0 rpm to 15.675 and 15.503 kWh at 5 and 10 rpm, respectively. At 60 °C, increasing the rotation speed from 0 to 5 and 10 rpm reduced total energy consumption from 14.400 to 10.120 and 9.350 kWh, respectively. At 70 °C, the corresponding total energy consumption decreased from 11.200 kWh at 0 rpm to 9.940 kWh at 5 rpm and 9.328 kWh at 10 rpm.

Although tray rotation required additional electrical energy, the reduced drying time decreased the energy consumed by the heater and blower. Consequently, total electrical energy consumption was lower under rotating conditions than under the stationary condition at each drying temperature. The lowest total electrical energy consumption was recorded at 70 °C and 10 rpm (9.328 kWh), closely followed by 60 °C and 10 rpm (9.350 kWh).

### 3.4. Drying Efficiency and Specific Energy Consumption

*η* and *SEC* differed among the nine drying conditions (Table 1 and Figure 4). *η* ranged from 26.87% at 50 °C and 0 rpm to 49.11% at 70 °C and 10 rpm. Conversely, SEC ranged from 4.60 to 8.40 MJ kg^−1^ water removed, with the highest value recorded at 50 °C and 0 rpm and the lowest value recorded at 70 °C and 10 rpm.

At 50 °C, *η* increased from 26.87% at 0 rpm to 29.25% at 5 rpm and 29.56% at 10 rpm; however, these values were not significantly different. *SEC* decreased significantly from 8.40 MJ kg^−1^ water removed at 0 rpm to 7.72 and 7.64 MJ kg^−1^ water removed at 5 and 10 rpm, respectively. No significant difference in *SEC* was detected between 5 and 10 rpm.

At 60 °C, *η* increased from 31.89% at 0 rpm to 45.33% at 5 rpm and 49.07% at 10 rpm. The corresponding *SEC* values decreased from 7.09 to 4.99 and 4.61 MJ kg^−1^ water removed. At 70 °C, *η* increased from 41.01% at 0 rpm to 46.24% at 5 rpm and 49.11% at 10 rpm, whereas *SEC* decreased from 5.52 to 4.90 and 4.60 MJ kg^−1^ water removed, respectively.

The treatments at 60 °C and 10 rpm and at 70 °C and 10 rpm produced statistically comparable energy performance; their drying efficiencies were 49.07% and 49.11%, respectively, while their *SEC* values were 4.61 and 4.60 MJ kg^−1^ water removed, respectively. At each drying temperature, the *SEC* and drying-efficiency values at 5 and 10 rpm did not differ significantly. Thus, increasing rotation speed from 5 to 10 rpm produced only a small numerical improvement in energy performance.

### 3.5. Factorial Effects of Drying-Air Temperature and Tray Rotation Speed

The two-way ANOVA results showed that drying-air temperature, tray rotation speed, and their interaction significantly affected drying time, *η*, and *SEC* (Table 2). Drying-air temperature produced the largest F-values for all three responses, indicating that temperature accounted for more variation than rotation speed. Its strongest effect was observed for drying time (F = 2630.600, *p* < 0.001), followed by *SEC* (F = 293.366, *p* < 0.001) and *η* (F = 193.433, *p* < 0.001). Tray rotation speed also significantly affected drying time (F = 96.200, *p* < 0.001), *η* (F = 57.011, *p* < 0.001), and *SEC* (F = 67.068, *p* < 0.001). However, its F- values were lower than those of drying-air temperature for all responses.

Significant temperature × rotation-speed interactions were detected for drying time (F = 11.300, *p* < 0.001), *η* (F = 12.065, *p* < 0.001), and *SEC* (F = 11.138, *p* < 0.001). These interactions demonstrate that the effect of tray rotation varied among drying-air temperatures, consistent with the treatment patterns presented in Figure 2, Figure 3 and Figure 4.

The factorial models explained 99.7%, 96.8%, and 97.7% of the observed variation in drying time, *η*, and *SEC*, respectively. Thus, the two experimental factors and their interaction adequately described the variation in the evaluated drying-performance responses.

### 3.6. Thin-Layer Model Performance

#### 3.6.1. Model Comparison

The nine thin-layer drying models were evaluated using the moisture-ratio data obtained from the six rotating-tray treatments at 5 and 10 rpm. Supplementary Table S4 presents the complete model parameters and goodness-of-fit statistics for these treatments. The stationary-tray treatments at 0 rpm were used as experimental controls for assessing the influence of tray rotation on drying process and energy responses and were not included in the detailed thin-layer model comparison. At 5 rpm, the Midilli model produced *R*^2^ values of 0.99865–0.99967, *RMSE* values of 0.00624–0.01075, and *χ*^2^ values of 0.00004–0.00012. Its best statistical performance was obtained at 70 °C, with *R*^2^ = 0.99967, *RMSE* = 0.00624, and *χ*^2^ = 0.00004.

At 10 rpm, the *R*^2^, *RMSE*, and *χ*^2^ values of the Midilli model ranged from 0.99865 to 0.99962, 0.00633 to 0.01169, and 0.00004 to 0.00014, respectively. The best fit at this rotation speed was obtained at 60 °C, with *R*^2^ = 0.99962, *RMSE* = 0.00633, and *χ*^2^ = 0.00004.

Among the evaluated models, the Midilli model provided the best overall fit for the six modeled rotating-tray treatments, as indicated by its consistently high *R*^2^ values and low *RMSE* and *χ*^2^ values. The model adequately represented the nonlinear reduction in moisture ratio at 50, 60, and 70 °C under tray rotation speeds of 5 and 10 rpm. Because the model comparison excluded the 0 rpm treatments, the selected model was not evaluated for the stationary-tray condition.

#### 3.6.2. Validation of the Selected Model

Figure 5 presents the relationship between experimental moisture-ratio values and those predicted by the Midilli model for the six rotating-tray treatments. The predicted values clustered near the 1:1 line across the evaluated drying-air temperatures and rotation speeds, demonstrating close agreement between the experimental and predicted data. This agreement was consistent with the high *R*^2^ and low *RMSE* and *χ*^2^ values reported in Supplementary Table S4.

The treatment-specific comparisons presented in Supplementary Figure S1 further showed that the Midilli-predicted moisture-ratio values closely followed the experimental values at 50, 60, and 70 °C under tray rotation speeds of 5 and 10 rpm. No substantial systematic deviation from the 1:1 relationship was evident over the investigated moisture-ratio range. These findings support using the Midilli model to describe moisture-ratio changes under the six modeled rotating-tray drying conditions. Because the predicted values were compared with the same experimental data used for parameter estimation, this assessment represents goodness of fit rather than independent external validation.

### 3.7. Apparent Effective Moisture Diffusivity and Activation Energy

#### 3.7.1. Apparent Effective Moisture Diffusivity

Apparent $D_{eff}$ was calculated using Equation (15). The resulting values ranged from 0.2457 × 10^−9^ to 1.2946 × 10^−9^ m^2^ s^−1^ and increased with both drying-air temperature and tray rotation speed (Figure 6a). The apparent $D_{eff}$ values for all nine experimental conditions, together with the corresponding Arrhenius regression parameters, are provided in Supplementary Table S3.

At 50 °C, apparent $D_{eff}$ increased from 0.2457 × 10^−9^ m^2^ s^−1^ at 0 rpm to 0.2847 × 10^−9^ and 0.2947 × 10^−9^ m^2^ s^−1^ at 5 and 10 rpm, respectively. At 60 °C, the corresponding values were 0.5980 × 10^−9^, 0.6818 × 10^−9^, and 0.7018 × 10^−9^ m^2^ s^−1^. The highest values were obtained at 70 °C, reaching 1.0822 × 10^−9^, 1.2254 × 10^−9^, and 1.2946 × 10^−9^ m^2^ s^−1^ at 0, 5, and 10 rpm, respectively.

At each drying-air temperature, the stationary-tray condition produced the lowest apparent $D_{eff}$, whereas 10 rpm produced the highest value. Increasing the rotation speed from 0 to 10 rpm increased apparent $D_{eff}$ by approximately 20.0%, 17.4%, and 19.6% at 50, 60, and 70 °C, respectively. The overall maximum apparent $D_{eff}$ was obtained at 70 °C and 10 rpm.

#### 3.7.2. Arrhenius Relationship and Activation Energy

The Arrhenius relationships between ln($D_{eff}$) and reciprocal absolute temperature (1000/T) are presented in Figure 6b. Linear relationships were obtained at all three rotation speeds, with *R*^2^ values ranging from 0.9905 to 0.9933. The resulting Arrhenius parameters are summarized in Table 3, while the complete regression equations and calculations are provided in Supplementary Table S3.

The apparent activation energies were 68.46, 67.39, and 68.32 kJ mol^−1^ at 0, 5, and 10 rpm, respectively (Table 3). The corresponding pre-exponential factors were 29.83, 23.20, and 33.73 m^2^ s^−1^. The similar activation-energy values indicate that tray rotation produced only a small change in the apparent temperature sensitivity of moisture diffusivity over the investigated temperature range.

## 4. Discussion

### 4.1. Temperature- and Rotation-Dependent Drying Behavior

Moisture ratio decreased continuously under all experimental conditions, with no identifiable constant-rate period. Drying therefore occurred predominantly in the falling-rate period, which is consistent with an increasing contribution of internal water migration after readily available surface moisture had evaporated. This behavior is common in biological materials because cellular structures and solid constituents restrict the movement of intracellular and bound water toward the surface [28,42]. Comparable falling-rate behavior and nonlinear moisture-ratio profiles have been reported for BSFL dried using convective and assisted-drying methods [17,43].

Drying-air temperature had a substantially stronger influence on drying behavior than tray rotation. Increasing the temperature from 50 to 70 °C shortened drying time by 74.2% without rotation, 74.5% at 5 rpm, and 75.5% at 10 rpm. This acceleration was consistent with greater temperature and vapor-pressure gradients between the larvae and drying air, which can enhance surface evaporation and internal moisture migration. Previous BSFL studies have similarly identified drying temperature and heating method as major determinants of drying rate and processing duration [17,43].

Tray rotation further reduced drying time. This reduction may be associated with repeated changes in the larvae’s position relative to the airflow and a corresponding decrease in external heat- and mass-transfer resistance. However, local airflow patterns and boundary-layer behavior were not measured directly. Relative to the stationary condition, rotation at 10 rpm reduced drying time by 14.5%, 37.5%, and 18.8% at 50, 60, and 70 °C, respectively. However, the comparatively small difference between 5 and 10 rpm suggests diminishing improvement in overall drying rate as the rotation speed increased beyond 5 rpm under the investigated conditions. It does not, however, confirm more uniform drying, because tray-to-tray and within-tray moisture distributions were not measured. The significant temperature × rotation-speed interaction further demonstrates that the effect of rotation depended on the thermal condition. Rotation produced its greatest relative reduction in drying time at 60 °C, whereas its contribution was more limited at 50 °C. This pattern suggests that the contribution of tray rotation depended on the combined thermal and external mass-transfer conditions rather than on rotation speed alone. The factorial interaction represents an important contribution because previous BSFL studies have mainly compared drying methods or evaluated temperature effects without explicitly examining their interaction with mechanical tray rotation [17,43,44].

### 4.2. Energy–Drying-Time Trade-Off

The heater contributed most to total electrical energy consumption, followed by the blower and tray-rotation motor. This distribution is consistent with convective drying, in which thermal-energy generation generally requires substantially more electricity than air circulation or low-speed mechanical movement. Drying is consequently recognized as an energy-intensive postharvest operation, and shortening processing time is an important strategy for reducing total energy demand [45].

Although tray rotation introduced an additional electrical load, its contribution was small relative to heater consumption. More importantly, rotation shortened heater and blower operation time. The resulting reductions in heater and blower energy consumption exceeded the additional electricity consumed by the rotation motor. This benefit was most evident at 60 °C, where rotation at 10 rpm reduced drying time and total electricity consumption by 37.5% and 35.1%, respectively. The smaller energy reductions at 50 and 70 °C show that the energy advantage of rotation depended on how much it shortened the drying process. Because drying-air velocity was maintained at 3.0 m s^−1^ across all treatments, the observed differences were associated with the investigated combinations of drying-air temperature and tray rotation speed rather than with changes in airflow velocity.

The 70 °C/10 rpm treatment was the best-performing tested condition for processing speed, producing the shortest drying time, lowest total electricity consumption, lowest *SEC*, highest *η*, and highest apparent $D_{eff}$. Nevertheless, the 60 °C/10 rpm treatment achieved statistically comparable *η* and *SEC* at a lower temperature, although its drying time was 3.5 h longer. Accordingly, 70 °C/10 rpm provided the most favorable measured combination of drying time and energy performance among the investigated treatments, whereas 60 °C/10 rpm represented a lower-temperature condition with comparable energy efficiency but a longer processing time. These comparisons are restricted to the measured engineering responses and do not establish which condition produced superior microbial safety or nutritional and physicochemical quality.

Drying temperature and exposure time can affect the microbiological characteristics, protein digestibility, lipid stability, color, and functional properties of BSFL products [17,44,46,47,48,49]. Previous studies have shown that thermal processing can reduce microbial populations, but the extent of inactivation depends on treatment temperature, exposure duration, pretreatment, initial microbial load, and processing configuration [46,48]. Other studies have shown that slaughter and drying conditions can also modify protein hydrolysis, in vitro digestibility, nutrient availability, and physicochemical properties [47,48,49]. Consequently, drying-air temperature, final moisture content, or energy performance alone cannot determine microbial lethality or product-quality retention.

### 4.3. Thin-Layer Modeling and Apparent Moisture Diffusivity

The Midilli model best represented the experimental moisture-ratio data for the six rotating-tray treatments at 5 and 10 rpm, with consistently high *R*^2^ and low *RMSE* and *χ*^2^ values. Its combination of an exponential component and a linear time-dependent term provides sufficient flexibility to represent rapid initial moisture removal followed by a progressively slower falling-rate period [30]. This model’s successful application aligns with its reported suitability for nonlinear drying curves in other agricultural materials [50].

Nevertheless, model suitability is material and process specific. Page, logarithmic, Wang and Singh, and other empirical models have successfully described the drying of rice, eggplant, leaves, and other biological products [51,52,53]. BSFL-specific studies have likewise demonstrated that model performance depends on drying temperature, heating method, pretreatment, and experimental configuration [17,43]. Accordingly, the Midilli model should be considered suitable within the investigated ranges of temperature, rotation speed, air velocity, and loading rather than universally applicable to all BSFL drying systems. Moreover, because the model comparison excluded the three stationary-tray treatments at 0 rpm, the selected model was not evaluated under stationary conditions.

The Midilli model could support estimating drying time and developing endpoint-control procedures for the rotating-tray dryer. However, it remains an empirical model: its good statistical fit does not prove a particular moisture-transfer mechanism, and its fitted constants should not be interpreted as fundamental transport properties. Moreover, the model represents the average system response. In practical multi-tray or thick-layer drying, a conventional thin-layer equation may not capture local temperature, airflow, and moisture gradients [54]. Because the experimental observations used to assess agreement were the same data used for parameter estimation, the comparison represents goodness of fit rather than independent external validation. Applying the model beyond the investigated conditions therefore requires validation with an independent dataset.

Apparent $D_{eff}$ increased from 0.2457 × 10^−9^ to 1.2946 × 10^−9^ m^2^ s^−1^ as temperature and rotation speed increased. The dominant temperature response is consistent with enhanced molecular mobility and a greater internal vapor-pressure gradient at higher temperatures. Similar temperature-dependent increases in apparent $D_{eff}$ have been reported for BSFL and other biological materials [17,43].

Tray rotation produced a more modest increase in apparent $D_{eff}$. Because $D_{eff}$ was derived from overall drying curves, it represents an apparent coefficient incorporating both internal and external transfer effects. The increase under rotating conditions should therefore be attributed cautiously to improved external air exposure and reduced boundary-layer resistance, rather than to a direct alteration of molecular diffusion within the larval tissues. Local airflow and boundary-layer characteristics were not measured directly; therefore, this proposed mechanism remains an interpretation of the observed drying behavior.

The apparent activation energies were similar at 0, 5, and 10 rpm, ranging from 67.39 to 68.46 kJ mol^−1^. This narrow range suggests that tray rotation modified external transfer conditions more strongly than the temperature sensitivity of internal moisture migration. Nevertheless, these values should be interpreted cautiously because each Arrhenius regression was based on only three temperatures and the diffusivity calculation assumed simplified geometry, constant diffusivity, and negligible shrinkage. Accordingly, the reported activation energies represent apparent estimates within the investigated temperature range rather than intrinsic material constants.

### 4.4. Engineering Implications for Rotating-Tray BSFL Drying

The 12 kg batch capacity provides a useful intermediate basis between small laboratory experiments and industrial BSFL processing. The results demonstrate that low-speed tray rotation can shorten drying time and reduce total electricity consumption despite the additional energy required by the drive motor. The main engineering benefit came from reducing the heater and blower operating time rather than lowering their instantaneous power demand. Nevertheless, the 12 kg batch capacity should not be regarded as industrial-scale validation, because the system was evaluated under a fixed loading, air velocity, and chamber configuration.

The Midilli model could be incorporated into process-control procedures to estimate moisture ratio and approximate the time required to reach the target moisture content. When combined with real-time measurements of sample mass, exhaust-air humidity, or drying-air temperature, it may support endpoint detection and reduce unnecessary dryer operation. However, such application would require independent model validation and evaluation under different batch loads, larval sizes, initial moisture contents, and airflow distributions.

Because electrical heating accounted for most of the system energy demand, future configurations could investigate waste heat, heat-pump systems, solar-assisted heating, or renewable electricity. Energy-efficient heat supply and improved process control are recognized as important directions for postharvest drying technology [45]. Nevertheless, the present results cannot infer their economic or environmental advantages without dedicated cost, exergy, and life-cycle analyses.

From an engineering perspective, the results indicate that tray rotation can shorten processing time and reduce total electricity consumption under the investigated conditions. However, selection of a drying condition for food or feed production must also consider microbial safety, water activity, nutrient retention, protein digestibility, lipid stability, and physicochemical properties [46,47,48,49]. Consequently, 70 °C/10 rpm should be regarded only as the best-performing investigated condition for drying time and energy use, whereas 60 °C/10 rpm represents a lower-temperature alternative with statistically comparable drying efficiency and specific energy consumption. No conclusion can be made regarding the relative microbial safety or product quality achieved under these two conditions.

### 4.5. Limitations and Future Research

The principal limitation of this study is that the experimental design did not include microbial and product-quality responses. We did not evaluate microbial counts, pathogen inactivation, water activity, protein quality, amino-acid availability, protein digestibility, nutrient retention, lipid oxidation, color, texture, or water-holding capacity. Previous studies have demonstrated that BSFL pretreatment and thermal processing can influence microbial populations, digestibility, nutrient availability, lipid stability, and physicochemical characteristics [17,44,46,47,48,49]. Consequently, these results cannot establish microbial safety, nutritional preservation, storage stability, regulatory compliance, or commercial suitability of dried BSFL.

The conditions evaluated in this study should therefore be interpreted as engineering-performance comparisons rather than an overall optimization for food or feed production. Although higher drying temperatures shortened drying time, their effects on protein functionality, digestibility, nutrient retention, and lipid oxidation remain unknown. Similarly, microbial lethality cannot be inferred solely from drying-air temperature, final moisture content, or processing time. Establishing a comprehensive process optimum will require simultaneous evaluation of drying time, energy consumption, microbial safety, nutritional retention, and physicochemical quality.

The findings are limited to an air velocity of 3.0 m s^−1^, three drying temperatures, three rotation speeds, and a fixed batch loading. Additional experiments should examine air velocity, loading density, layer thickness, and intermittent rotation to determine whether equivalent drying performance can be achieved with lower auxiliary energy input. Additional temperature levels would also strengthen the Arrhenius analysis and better characterize the temperature dependence of apparent moisture diffusivity.

Tray-to-tray and within-tray moisture distributions were not measured directly. Thus, although rotation shortened overall drying time, the study does not demonstrate improved drying uniformity. Future research should quantify moisture variation at defined radial and vertical locations and measure airflow and temperature distributions throughout the chamber. This is particularly important for multi-tray systems because average drying curves may conceal localized moisture gradients [54].

The infinite-slab representation also simplified the irregular geometry of individual larvae. We assumed constant diffusivity and negligible shrinkage, although biological tissues may contract and undergo structural changes during drying. Direct measurement of larval dimensions and shrinkage, combined with finite-geometry or numerical diffusion models, would improve the physical interpretation of moisture transport. Therefore, the calculated $D_{eff}$ values should be interpreted as apparent coefficients that describe moisture-transfer behavior under the stated assumptions.

A conventional oven or an alternative commercial dryer was not included as an external benchmark. The 0 rpm treatment served as the internal stationary-tray control within the same drying chamber, heating system, airflow condition, loading arrangement, and measurement procedure. This design isolated the effect of tray rotation but did not establish that the developed system outperformed conventional or commercial dryers. A direct technology comparison would require a separate experiment with harmonized loading, endpoint, airflow, and energy-accounting conditions.

Future work should integrate microbiological and product-quality responses with drying time, energy performance, and moisture uniformity. Exergy, economic, and life-cycle analyses are also required before assessing commercial-scale implementation.

## 5. Conclusions

This study evaluated the drying kinetics, energy performance, and apparent moisture-transport characteristics of BSFL in a 12 kg rotating-tray hot-air dryer using a 3 × 3 factorial design comprising drying-air temperatures of 50, 60, and 70 °C and tray rotation speeds of 0, 5, and 10 rpm. Drying-air temperature was the dominant factor affecting drying time, drying efficiency, and specific energy consumption, while tray rotation further improved performance by shortening the required operating time and reducing heater and blower energy consumption. A significant interaction between temperature and rotation speed indicated that the effectiveness of rotation depended on the applied drying temperature.

The shortest drying time (6.5 h) was obtained at 70 °C and 10 rpm, compared with 31.0 h at 50 °C without rotation. The 70 °C and 10 rpm condition also produced the highest *η* (49.11%), the lowest *SEC* (4.60 MJ kg^−1^ water removed), and the highest apparent $D_{eff}$. However, drying at 60 °C and 10 rpm achieved statistically comparable energy performance, with an *η* of 49.07% and an *SEC* of 4.61 MJ kg^−1^ water removed, although the drying time increased to 10.0 h. Among the nine thin-layer models evaluated for the six rotating-tray treatments at 5 and 10 rpm, the Midilli model best fit the experimental moisture-ratio data. Apparent $D_{eff}$ increased from 0.2457 × 10^−9^ to 1.2946 × 10^−9^ m^2^ s^−1^, while the apparent activation energy ranged from 67.39 to 68.46 kJ mol^−1^ across the three rotation speeds.

Among the investigated treatments, 70 °C and 10 rpm provided the most favorable combination of drying time and energy performance, whereas 60 °C and 10 rpm represented a lower-temperature alternative with statistically comparable drying efficiency and specific energy consumption. These findings identify favorable engineering conditions within the investigated range but do not establish an overall optimum for food or feed production. Microbial lethality, water activity, protein digestibility, nutrient retention, lipid oxidation, and physicochemical quality were not evaluated. Consequently, the investigated conditions should not be interpreted as confirming microbial safety, nutritional preservation, storage stability, or commercial suitability of the dried BSFL. Future research should integrate these product-quality responses with drying kinetics, energy performance, and moisture uniformity to establish a comprehensive multi-response process optimum.

## Figures and Tables

**Figure 1:**
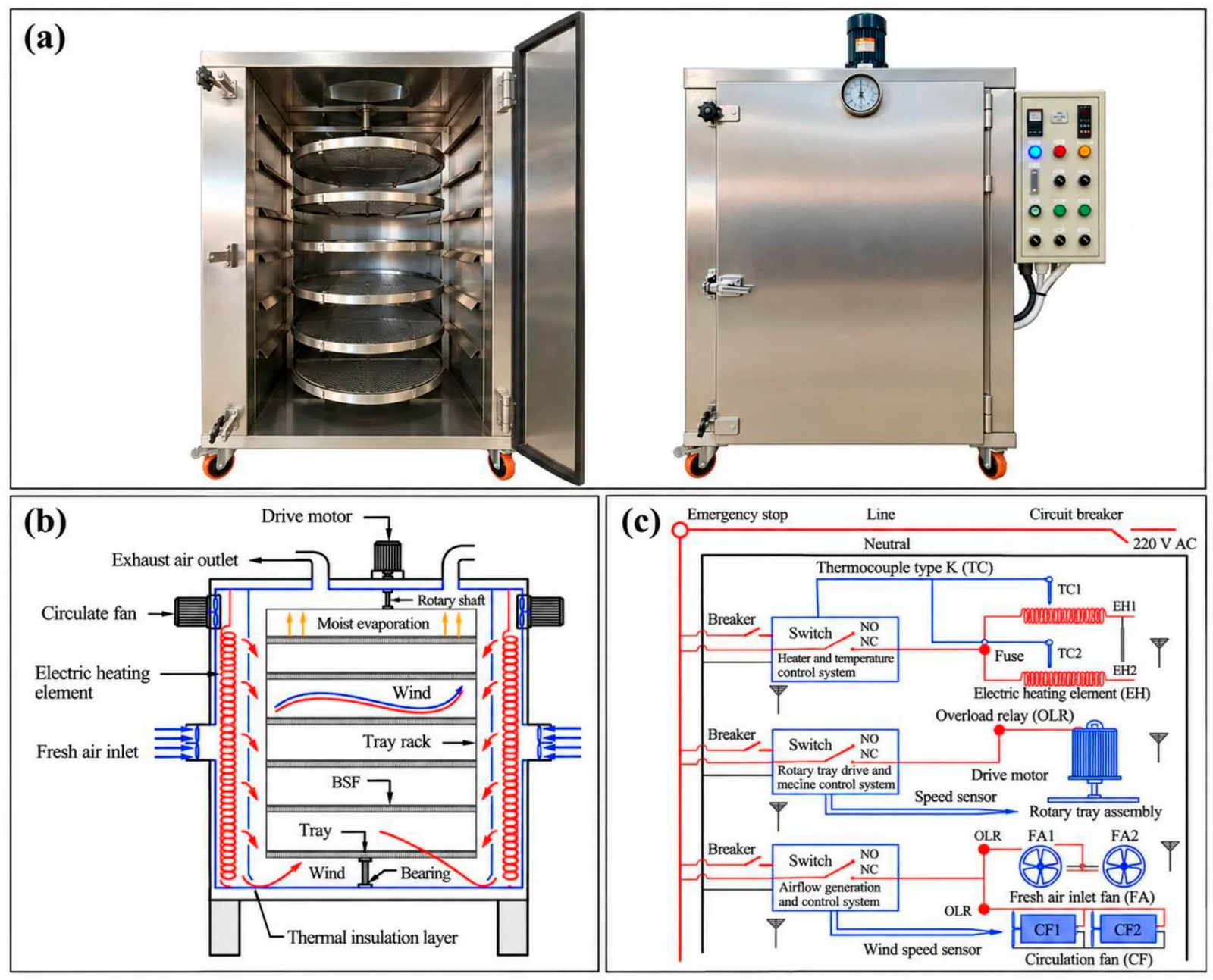
Rotating-tray hot-air drying system: (**a**) photographs of the experimental dryer showing the open drying chamber, tray assembly, closed chamber, and control panel; (**b**) blue arrows indicate incoming and circulating airflow, whereas red arrows indicate heated-air movement; and (**c**) red lines indicate power wiring and blue lines indicate control and sensor circuits.

**Figure 2:**
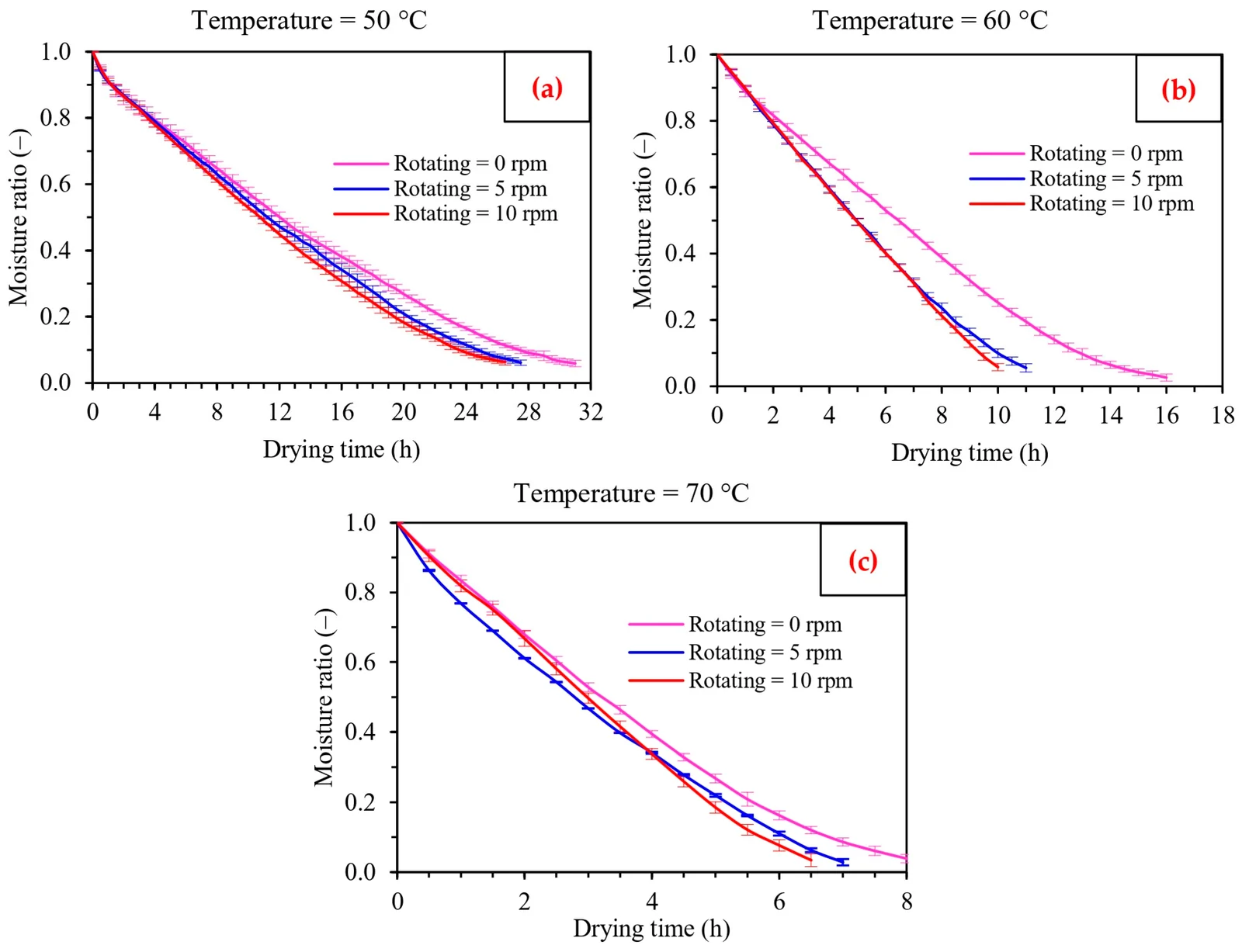
Moisture-ratio profiles of BSFL during rotating-tray hot-air drying at rotation speeds of 0, 5, and 10 rpm: (**a**) 50 °C; (**b**) 60 °C; and (**c**) 70 °C. Each plotted value represents the mean of three independent experimental runs, and the error bars indicate ±SD.

**Figure 3:**
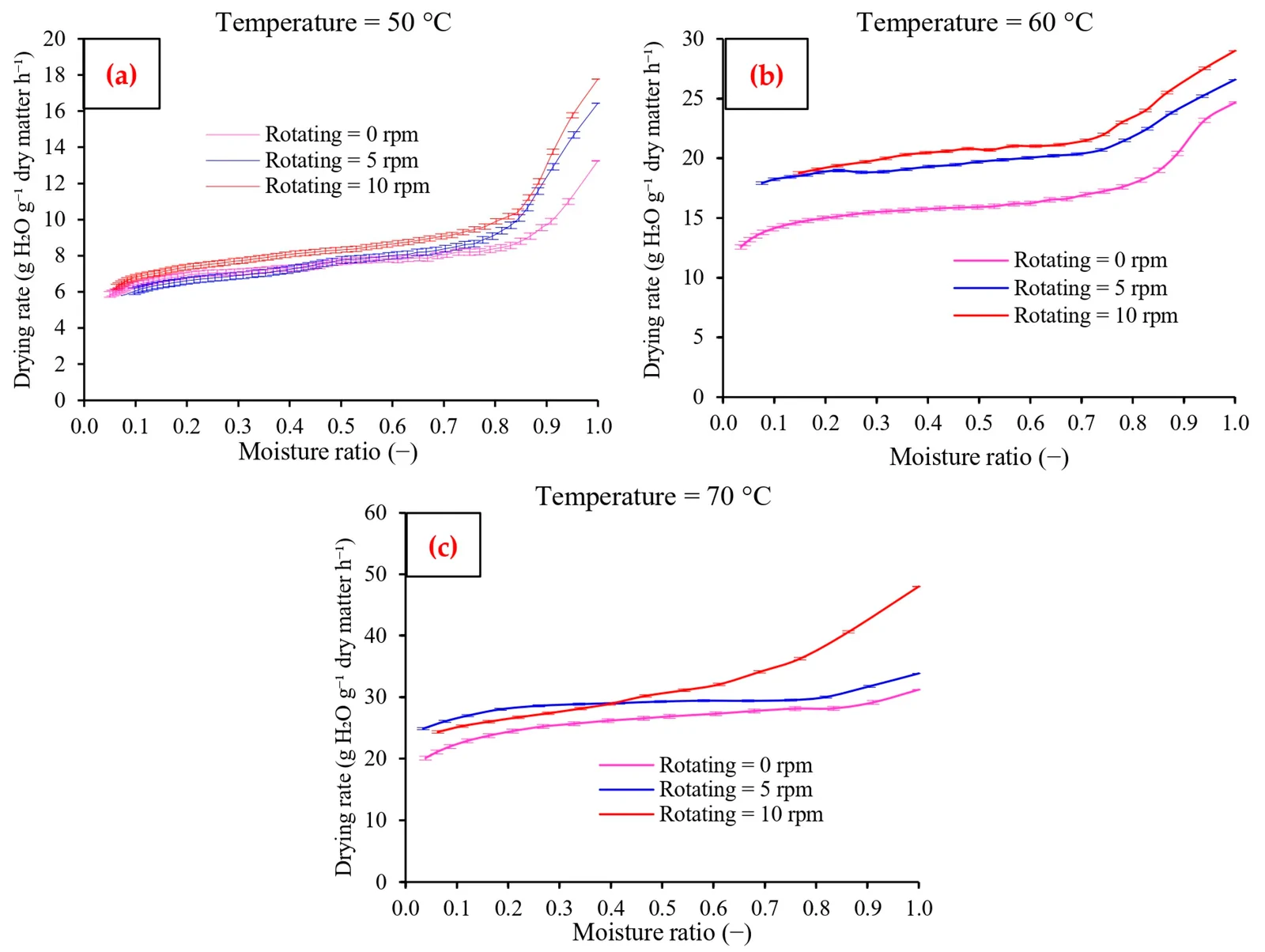
Drying rate as a function of moisture ratio for BSFL during rotating-tray hot-air drying at rotation speeds of 0, 5, and 10 rpm: (**a**) 50 °C; (**b**) 60 °C; and (**c**) 70 °C. Data points represent the means of three independent experimental runs, and error bars indicate ±SD.

**Figure 4:**
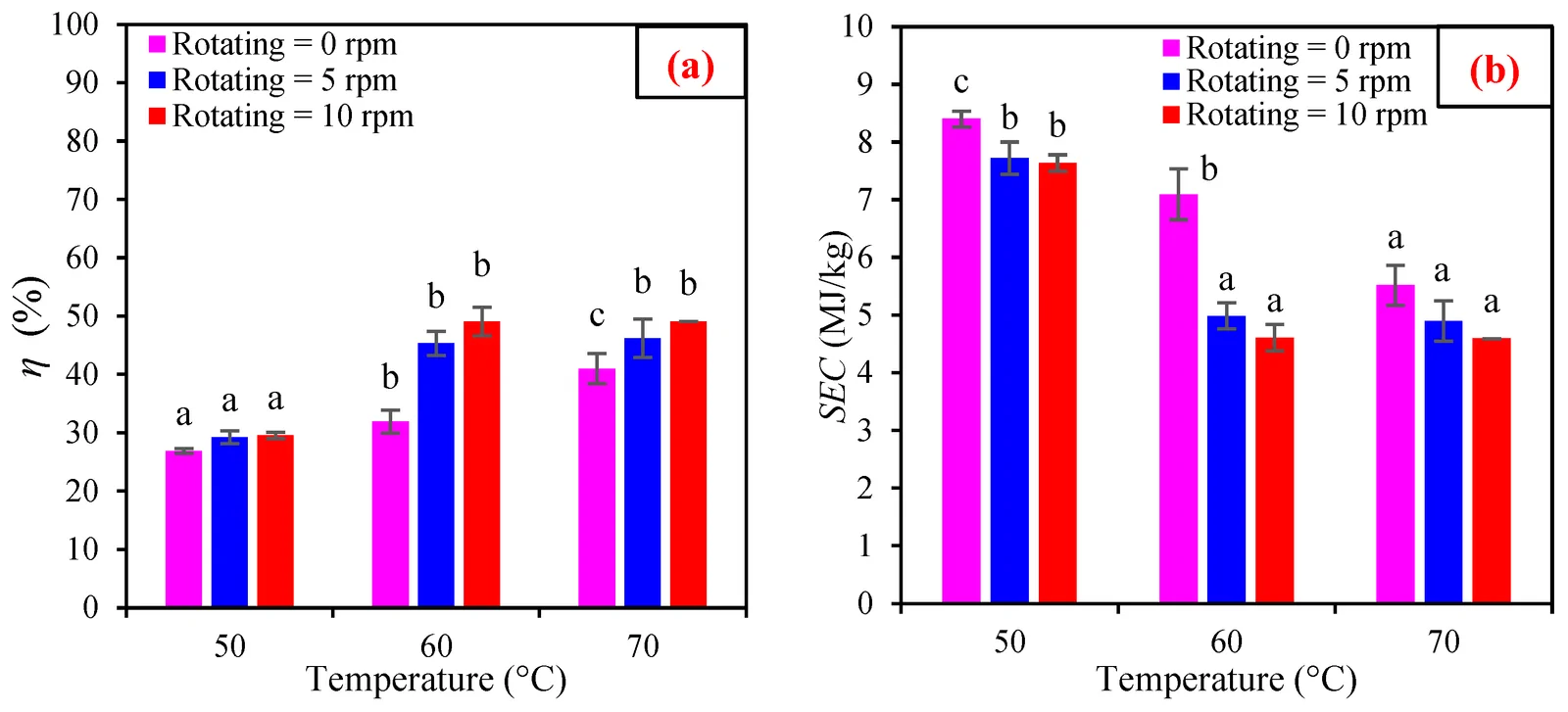
Effects of drying-air temperature and tray rotation speed on (**a**) drying efficiency, *η* (%) and (**b**) specific energy consumption (*SEC*) during rotating-tray hot-air drying of BSFL. Bars represent mean ± SD (n = 3). Different lowercase letters above the bars indicate significant differences among treatment means within each response according to Tukey’s HSD test (*p* < 0.05).

**Figure 5:**
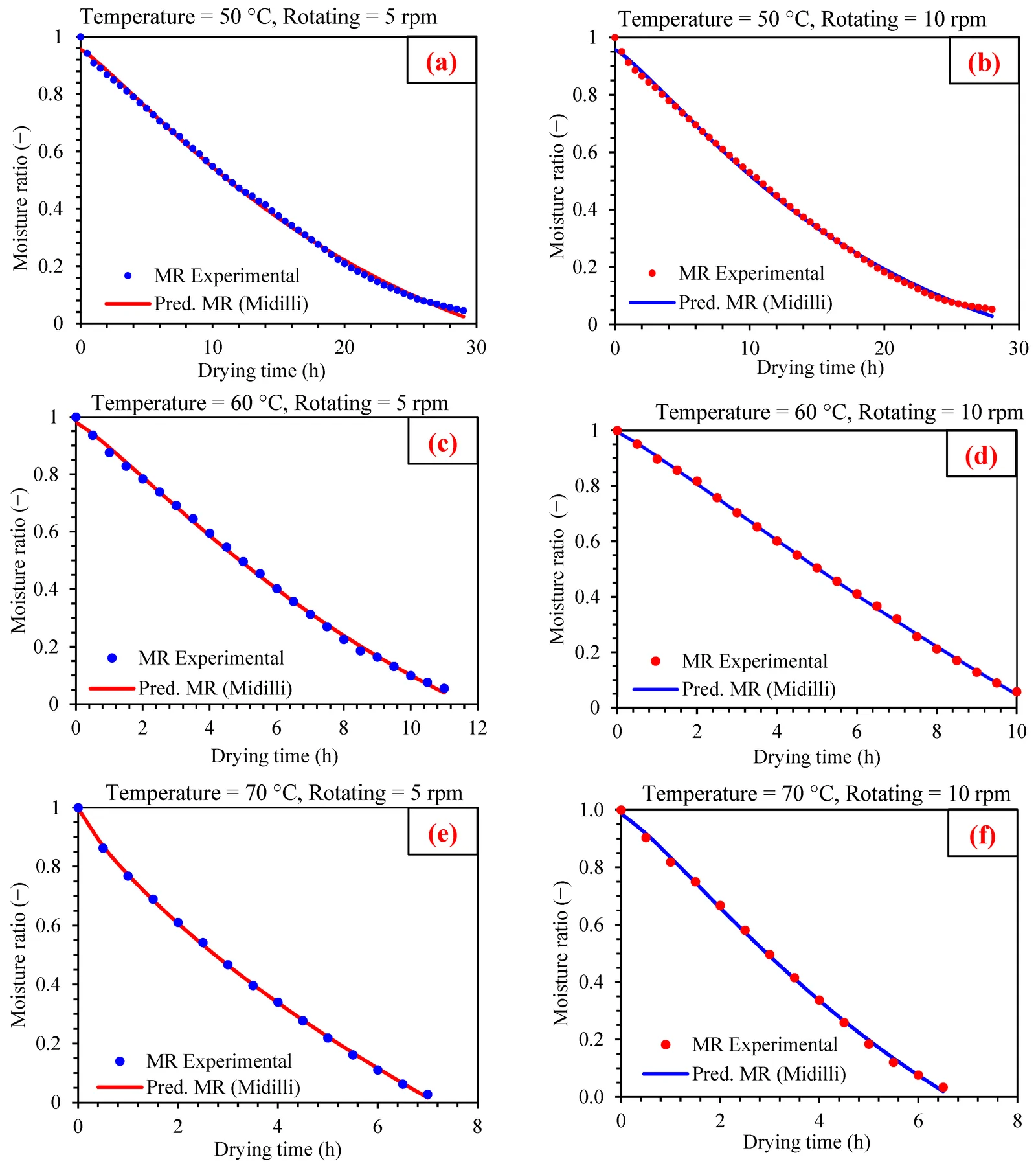
Experimental moisture-ratio values and Midilli-model predictions over drying time for BSFL under six rotating-tray drying conditions: (**a**) 50 °C and 5 rpm; (**b**) 50 °C and 10 rpm; (**c**) 60 °C and 5 rpm; (**d**) 60 °C and 10 rpm; (**e**) 70 °C and 5 rpm; and (**f**) 70 °C and 10 rpm. Symbols represent experimental values, and lines represent Midilli-model predictions.

**Figure 6:**
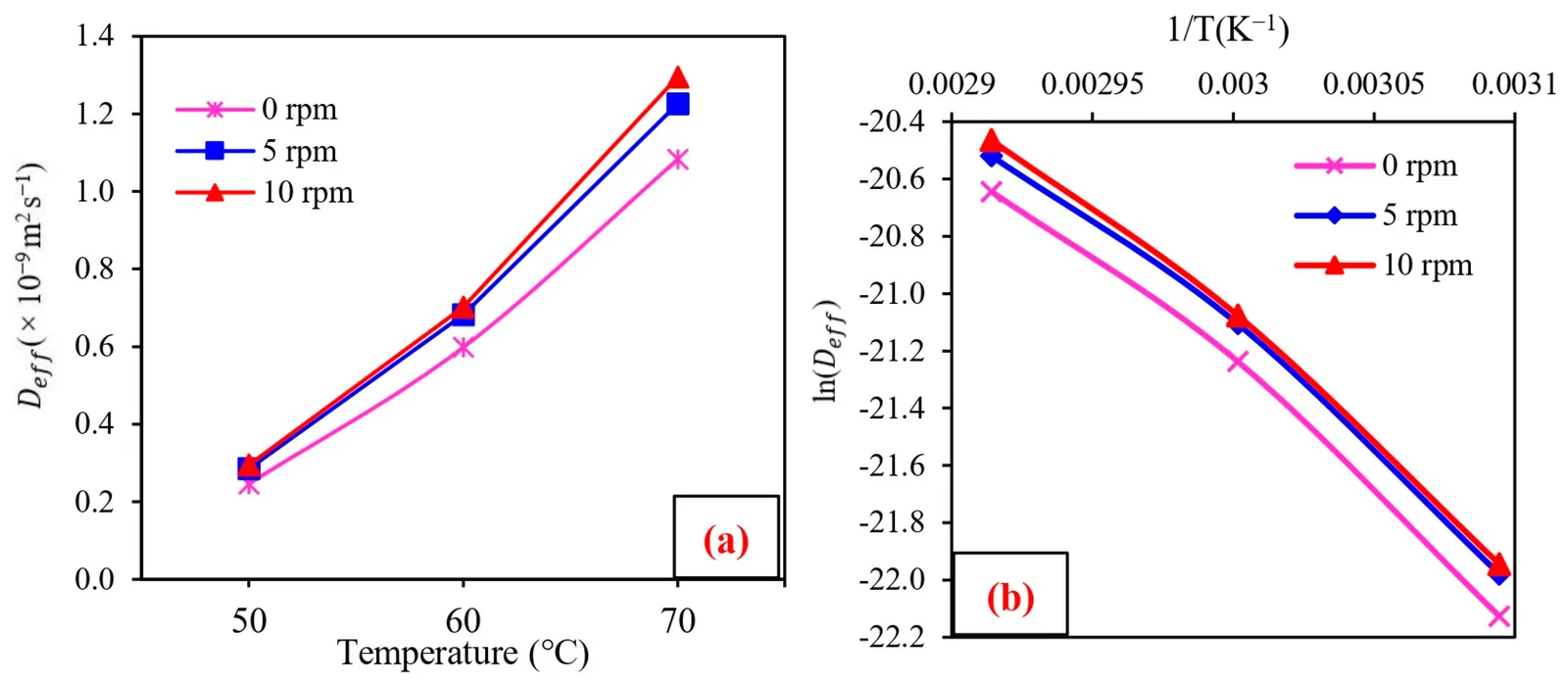
Effects of drying-air temperature and tray rotation speed on moisture transport in BSFL: (**a**) effective moisture diffusivity ($D_{eff})$ at 50, 60, and 70 °C; and (**b**) Arrhenius relationships between ln($D_{eff}$) and reciprocal absolute temperature (1000/T) at 0, 5, and 10 rpm.

**Table 1:** Energy consumption and performance of BSFL under different drying-air temperatures and tray rotation speeds.

Drying Conditions			Electrical Energy Consumption (kWh)				Energy Performance	
Temperature (°C)	Rotation Speed (rpm)	Drying Time (h)	($E_{b}$)	($E_{h}$)	($E_{r}$)	($E_{total}$)	*SEC* (MJ kg^−1^ Water Removed)	*η* (%)
50	0	31.0	4.650	12.400	0.000	17.050	8.40 ^d^	26.87 ^a^
50	5	27.5	4.125	11.000	0.550	15.675	7.72 ^c^	29.25 ^ab^
50	10	26.5	3.975	10.600	0.928	15.503	7.64 ^c^	29.56 ^ab^
60	0	16.0	2.400	12.000	0.000	14.400	7.09 ^c^	31.89 ^b^
60	5	11.0	1.650	8.250	0.220	10.120	4.99 ^ab^	45.33 ^cd^
60	10	10.0	1.500	7.500	0.350	9.350	4.61 ^a^	49.07 ^d^
70	0	8.0	1.200	10.000	0.000	11.200	5.52 ^b^	41.01 ^c^
70	5	7.0	1.050	8.750	0.140	9.940	4.90 ^ab^	46.24 ^d^
70	10	6.5	0.975	8.125	0.228	9.328	4.60 ^a^	49.11 ^d^

Note: Different superscript letters within the same column indicate significant differences among treatment means according to Tukey’s honestly significant difference test at (*p* < 0.05). *SEC*, specific energy consumption.

**Table 2:** Two-way ANOVA results for the effects of drying-air temperature and tray rotation speed on the drying performance of BSFL.

Dependent Variable	Source of Variation	df	*F*-Value	*p*-Value
Drying time (h)	Temperature	2	2630.600	*p* < 0.0001
*R*^2^ = 0.997	Tray rotation speed	2	96.200	*p* < 0.0001
	Temperature × Tray rotation speed	4	11.300	*p* < 0.0001
	Error	18		
*η* (%)	Temperature	2	193.433	*p* < 0.0001
*R*^2^ = 0.968	Tray rotation speed	2	57.011	*p* < 0.0001
	Temperature × Tray rotation speed	4	12.065	*p* < 0.0001
	Error	18		
*SEC* (MJ kg^−1^ water removed)	Temperature	2	293.366	*p* < 0.0001
*R*^2^ = 0.977	Tray rotation speed	2	67.068	*p* < 0.0001
	Temperature × Tray rotation speed	4	11.138	*p =* 0.0001
	Error	18		

Note: *SEC*, specific energy consumption. Treatment means were compared using Tukey’s honestly significant difference test at (*p* < 0.05).

**Table 3:** Arrhenius parameters for apparent $D_{eff}$ of BSFL at different tray rotation speeds.

Tray Rotation Speed (rpm)	$E_{a}$ (kJ mol^−1^)	$D_{0}$ (m^2^ s^−1^)	*R* ^2^
0	68.46	29.83	0.9905
5	67.39	23.20	0.9909
10	68.32	33.73	0.9933

## Data Availability

Data will be made available upon reasonable request.

## Supplementary Materials

The following supporting information can be downloaded at https://www.mdpi.com/article/10.3390/insects17100984/s1: Figure S1: Experimental and Midilli-predicted moisture ratios under six rotating-tray drying conditions; Table S1: Dryer equipment and instrument specifications; Table S2: Thin-layer drying models; Table S3: Apparent effective moisture diffusivity and Arrhenius parameters; Table S4: Thin-layer model parameters and goodness-of-fit statistics.
