# Supplementary material for: Drying Kinetics, Apparent Moisture Diffusivity, and Energy Performance of Black Soldier Fly Larvae in a Rotating-Tray Hot-Air Dryer
Source: Insects. 2026 Sep 23;17(10):984. doi: 10.3390/insects17100984 (PMC13705072; doi:10.3390/insects17100984)
Supplement: Supplementary file 1 [file insects-17-00984-s001.zip › insects-4521507-supplementary.pdf]

**Table S1.** Specifications and measurement characteristics of the equipment and instruments used in the rotating-tray hot-air drying experiments.

| Equipment or instrument | Manufacturer/model | Measurement or operating range | Accuracy or rated specification | Measured variable or function |
|---|---|---|---|---|
| Electronic balance | OHAUS PA4102 (Ohaus, Parsippany, NJ, USA) | 0.1–4100 g | ±0.01 g | Sample mass |
| Electric heating unit | Custom-built finned-tube heater | 30–120 °C | Temperature-control accuracy: ±1 °C | Heating and regulation of drying air |
| Hot-wire anemometer | UNI-T UT363S (Uni-Trend Technology Co., Ltd., Dongguan, Guangdong, China) | 0.4–30 m $s^{-1}$ | ±(5% of reading + 0.5 m $s^{-1}$) | Drying-air velocity |
| Digital clamp meter | KYORITSU IEC61010-2-031 (Kyoritsu, Tokyo, Japan) | 0–400 A | Current resolution: ±0.01 A* | Electrical consumption of heater, blower, and tray motor |
| K-type thermocouple | Type K | −50 to 1250 °C | ±1.5 °C | Drying-air temperature |
| Laboratory drying oven | Memmert UN30 (Memmert, Schwabach, Germany) | 20–300 °C | ±0.10 °C | Initial and final moisture-content determination |
| Multichannel data logger | BTM-4208SD (Lutron Electronic Enterprise Co., Ltd., Taipei, Taiwan) | −50 to 999.9 °C | ±(0.4% of reading + 0.5 °C) | Acquisition of thermocouple measurements |

**Supplementary Table S2.** Thin-layer drying models used to describe the drying kinetics of black soldier fly larvae.

| No. | Model | Equation | Reference |
|---|---|---|---|
| **1** | Midilli | $MR = a\ exp(-kt^n) + bt$ | [30] |
| **2** | Wang and Singh | $MR = 1 + at + bt^2$ | [31] |
| **3** | Approximation of diffusion | $MR = a\ exp(-kt) + (1 - a)\ exp(-k_b t)$ | [32] |
| **4** | Page | $MR = exp(-kt^n)$ | [33] |
| **5** | Logarithmic | $MR = a\ exp(-kt) + c$ | [34] |
| **6** | Two-term | $MR = a\ exp(-kt) + b\ exp(-k_1 t)$ | [35] |
| **7** | Modified Henderson and Pabis | $MR = a\ exp(-kt) + b\ exp(-gt) + c\ exp(-ht)$ | [36] |
| **8** | Henderson and Pabis | $MR = a\ exp(-kt)$ | [37] |
| **9** | Newton | $MR = exp(-kt)$ | [38] |

Note: $MR$ is the dimensionless moisture ratio; $t$ is drying time (h); $k$, $k_1$ , $g$, and $h$ are drying-rate constants; and $a$, $b$, $c$, and $n$ are model coefficients or exponents. The units of the drying-rate constants depend on the mathematical form of the corresponding model.

**Supplementary Table S3.** Effective moisture diffusivity and Arrhenius regression parameters for black soldier fly larvae under different drying-air temperatures and tray rotation speeds.

**Part A.** Effective moisture diffusivity

| Drying-air temperature (°C) | Absolute temperature (K) | Tray rotation speed (rpm) | $D_{eff}$ (×$10^{-9}$ m$^2$ s$^{-1}$) |
|---|---|---|---|
| 50 | 323.15 | 0 | 0.2457 |
| 50 | 323.15 | 5 | 0.2847 |
| 50 | 323.15 | 10 | 0.2947 |
| 60 | 333.15 | 0 | 0.5980 |
| 60 | 333.15 | 5 | 0.6818 |
| 60 | 333.15 | 10 | 0.7018 |
| 70 | 343.15 | 0 | 1.0822 |
| 70 | 343.15 | 5 | 1.2254 |
| 70 | 343.15 | 10 | 1.2946 |

**Part B.** Arrhenius regression parameters

| Rotation speed (rpm) | Arrhenius regression equation | $D_0$ (m$^2$ s$^{-1}$) | $E_a$ (kJ mol$^{-1}$) | $R^2$ |
|---|---|---|---|---|
| **0** | $\ln(D_{eff})$ = 3.3954–8234.38(1/T) | 29.83 | 68.46 | 0.9905 |
| **5** | $\ln(D_{eff})$ = 3.1443–8106.09(1/T) | 23.20 | 67.39 | 0.9909 |
| **10** | $\ln(D_{eff})$ = 3.5183–8217.47(1/T) | 33.73 | 68.32 | 0.9933 |

Note: $D_{eff}$ = effective moisture diffusivity; $D_0$ = pre-exponential factor; $E_a$ = apparent activation energy; and T = absolute drying-air temperature.

**Supplementary Table S4.** Estimated parameters and goodness-of-fit statistics of nine thin-layer drying models for BSFL under six rotating-tray drying conditions at 5 and 10 rpm.

| **Model** | **Temp. (◦C)** | **Coefficient and constant** | ***R***$^2$ | ***RMSE*** | $\chi^2$ |
|---|---|---|---|---|---|
| | | **Rotating = 5 rpm** | | | |
| **1** | 50 | a = 0.95595, k = 0.02977, n = 1.19415, b = −0.00544 | 0.99865 | 0.01075 | 0.00012 |
| | 60 | a = 0.98173, k = 0.07205, n = 1.20863, b = −0.02050 | 0.99895 | 0.01038 | 0.00011 |
| | 70 | a = 0.99706, k = 0.18784, n = 0.82532, b = −0.05352 | 0.99967 | 0.00624 | 0.00004 |
| **2** | 50 | a = −0.05190, b = 0.00063 | 0.99811 | 0.01259 | 0.00035 |
| | 60 | a = −0.11350, b = 0.00235 | 0.99886 | 0.01056 | 0.00021 |
| | 70 | a = −0.20968, b = 0.01029 | 0.99734 | 0.01690 | 0.00052 |
| **3** | 50 | a = 1.62590, k = 0.03466, $k_b$ = 0.00295 | 0.99794 | 0.01314 | 0.00017 |
| | 60 | a = 4.40120, k = 0.03868, $k_b$ = 0.01629 | 0.99867 | 0.01140 | 0.00013 |
| | 70 | a = 3.08430, k = 0.11063, $k_b$ = 0.05790 | 0.99822 | 0.01381 | 0.00019 |
| **4** | 50 | k = 0.03138, n = 1.30160 | 0.98968 | 0.02915 | 0.00088 |
| | 60 | k = 0.07471, n = 1.43411 | 0.99163 | 0.02795 | 0.00086 |
| | 70 | k = 0.20591, n = 1.26071 | 0.98689 | 0.03604 | 0.00153 |
| **5** | 50 | a = 1.58686, k = 0.03233, c = −0.60566 | 0.99837 | 0.01170 | 0.00014 |
| | 60 | a = 2.21871, k = 0.05218, c = −1.21735 | 0.99864 | 0.01155 | 0.00013 |
| | 70 | a = 1.59600, k = 0.12964, c = −0.61782 | 0.99904 | 0.01013 | 0.00010 |
| **6** | 50 | a = 2.78422, b = −1.80354, k = 0.02518, $k_1$ = −0.01065 | 0.99845 | 0.01149 | 0.00013 |
| | 60 | a = 1.77770, b = −0.77597, k = 0.07205, $k_1$ = 0.01186 | 0.99859 | 0.01204 | 0.00015 |
| | 70 | a = 1.51814, b = −0.54043, k = 0.13328, $k_1$ = 0.00800 | 0.99905 | 0.01053 | 0.00011 |
| **7** | 50 | a = 0.03900, b = 1.00902, c = 0.03240, k = 0.07690, g = 0.07690, h = 0.07690 | 0.97360 | 0.04834 | 0.00264 |
| | 60 | a = 0.00844, b = 1.14379, c = 0.01048, k = 0.20235, g = 0.19214, h = 0.18552 | 0.97124 | 0.05757 | 0.00512 |
| | 70 | a = 0.00698, b = 1.13249, c = 0.00826, k = 0.33173, g = 0.33169, h = 0.33165 | 0.96600 | 0.06974 | 0.01459 |
| **8** | 50 | a = 1.05558, k = 0.07503 | 0.97182 | 0.04816 | 0.00232 |
| | 60 | a = 1.07817, k = 0.17738 | 0.96384 | 0.05808 | 0.00337 |
| | 70 | a = 1.03924, k = 0.30119 | 0.97359 | 0.05114 | 0.00262 |
| **9** | 50 | k = 0.07074 | 0.96716 | 0.05153 | 0.00266 |
| | 60 | k = 0.16291 | 0.95366 | 0.06424 | 0.00413 |
| | 70 | k = 0.28894 | 0.97099 | 0.05165 | 0.00267 |

| **Model** | **Temp. (◦C)** | **Coefficient and constant** | ***R***$^2$ | ***RMSE*** | $\chi^2$ |
|---|---|---|---|---|---|
| | | **Rotating = 10 rpm** | | | |
| **1** | 50 | a = 0.95767, k = 0.03189, n = 1.22032, b = −0.00428 | 0.99865 | 0.01076 | 0.00012 |
| | 60 | a = 0.99524, k = 0.04995, n = 1.20557, b = −0.03981 | 0.99962 | 0.00633 | 0.00004 |
| | 70 | a = 0.98743, k = 0.12184, n = 1.24579, b = −0.04032 | 0.99898 | 0.01169 | 0.00014 |
| **2** | 50 | a = −0.05553, b = 0.00075 | 0.99854 | 0.01112 | 0.00028 |
| | 60 | a = −0.10153, b = 0.00056 | 0.99935 | 0.00806 | 0.00011 |
| | 70 | a = −0.18397, b = 0.00491 | 0.99858 | 0.01310 | 0.00028 |
| **3** | 50 | a = 3.72158, k = 0.02921, $k_b$ = 0.01854 | 0.99812 | 0.01259 | 0.00016 |
| | 60 | a = 8.73957, k = 0.01162, $k_b$ = < 0.0001 | 0.99934 | 0.00810 | 0.00006 |
| | 70 | a = 4.67799, k = 0.05063, $k_b$ = 0.01409 | 0.99845 | 0.01369 | 0.00019 |

**Supplementary Table S4.** Estimated parameters and goodness−of−fit statistics of nine thin−layer drying models for BSFL under six rotating−tray drying conditions at 5 and 10 rpm (*Cont.*).

| **Model** | **Temp. (◦C)** | **Coefficient and constant** | ***$R^2$*** | ***RMSE*** | ***$\chi^2$*** |
|---|---|---|---|---|---|
| | | **Rotating = 10 rpm** | | | |
| **4** | 50 | k = 0.03453, n = 1.29259 | 0.99184 | 0.02602 | 0.00070 |
| | 60 | k = 0.06000, n = 1.55499 | 0.99050 | 0.02994 | 0.00100 |
| | 70 | k = 0.13931, n = 1.53718 | 0.99037 | 0.03271 | 0.00128 |
| **5** | 50 | a = 1.46874, k = 0.03859, c = −0.48025 | 0.99817 | 0.01244 | 0.00015 |
| | 60 | a = 4.79955, k = 0.02241, c = −3.78729 | 0.99935 | 0.00803 | 0.00006 |
| | 70 | a = 2.80556, k = 0.06739, c = −1.79962 | 0.99848 | 0.01355 | 0.00018 |
| **6** | 50 | a = 3.11994, b = −2.13211, k = 0.02854, $k_1$ = −0.01546 | 0.99831 | 0.01206 | 0.00015 |
| | 60 | a = 2.36012, b = −1.35227, k = 0.03297, $k_1$ = 0.02020 | 0.99940 | 0.00794 | 0.00006 |
| | 70 | a = 2.25919, b = −1.25310, k = 0.07697, $k_1$ = 0.01250 | 0.99846 | 0.01434 | 0.00021 |
| **7** | 50 | a = 0.03893, b = 1.01146, c = 0.03235, k = 0.08155, g = 0.08155, h = 0.08155 | 0.97711 | 0.04526 | 0.00232 |
| | 60 | a = 0.00844, b = 1.18087, c = 0.01047, k = 0.19563, g = 0.19561, h = 0.19561 | 0.96525 | 0.06444 | 0.00692 |
| | 70 | a = 0.00698, b = 1.23424, c = 0.00826, k = 0.34295, g = 0.34292, h = 0.34290 | 0.96580 | 0.07551 | 0.02281 |
| **8** | 50 | a = 1.05749, k = 0.07955 | 0.97533 | 0.04525 | 0.00205 |
| | 60 | a = 1.09617, k = 0.17707 | 0.95208 | 0.06723 | 0.00452 |
| | 70 | a = 1.08359, k = 0.30286 | 0.95331 | 0.07204 | 0.00519 |
| **9** | 50 | k = 0.07020 | 0.96930 | 0.04998 | 0.00250 |
| | 60 | k = 0.15873 | 0.93555 | 0.07600 | 0.00578 |
| | 70 | k = 0.27738 | 0.94182 | 0.07726 | 0.00597 |

Note*:* Models 1–9 correspond to the Midilli, Wang and Singh, approximation of diffusion, Page, logarithmic, two−term, modified Henderson and Pabis, Henderson and Pabis, and Newton models, respectively, as defined in Supplementary Table S2. Model selection was based on the highest $R^2$ and the lowest *RMSE* and $\chi^2$ values.

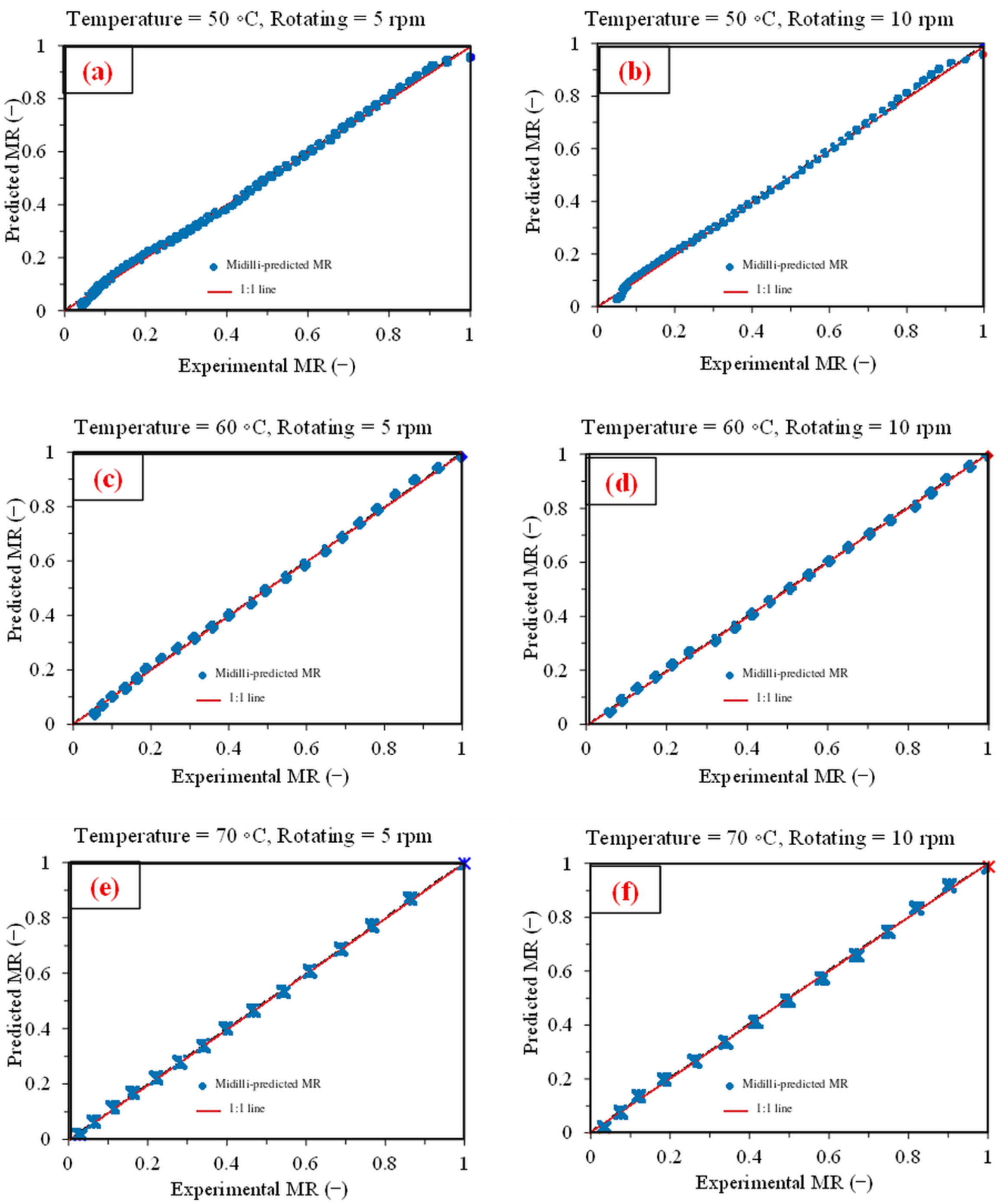


**Supplementary Figure S1.** Treatment−specific relationships between experimental and Midilli model−predicted moisture ratios for BSFL under rotating−tray hot−air drying: (**a**) 50 °C and 5 rpm; (**b**) 50 °C and 10 rpm; (**c**) 60 °C and 5 rpm; (**d**) 60 °C and 10 rpm; (**e**) 70 °C and 5 rpm; and (**f**) 70 °C and 10 rpm. The solid red lines represent the exact 1:1 relationship.
